# Monotherapy With Immune Checkpoint Inhibitors in Patients With Recurrent and/or Metastatic Sinonasal Squamous Cell Carcinoma

**DOI:** 10.1002/cam4.71391

**Published:** 2025-11-27

**Authors:** Alexander Lein, Thorsten Fuereder, Manuel Stoeth, Agmal Scherzad, Stephan Hackenberg, Julia Schnöll, Lorenz Kadletz‐Wanke, Gregor Heiduschka, Archana Jaiswal, Rajiv Bhalla, Lukas Kenner, Faris F. Brkic

**Affiliations:** ^1^ Department of Otorhinolaryngology, Head and Neck Surgery Medical University of Vienna Vienna Austria; ^2^ Division of Oncology, Department of Medicine I Medical University of Vienna Vienna Austria; ^3^ Department of Otorhinolaryngology, Head and Neck Surgery University Hospital Würzburg Würzburg Germany; ^4^ Department of Otorhinolaryngology Manchester University NHS Foundation Trust Manchester UK; ^5^ Department of Otorhinolaryngology Manchester Centre for Clinical Neurosciences, Northern Care Alliance NHS Foundation Trust Salford UK; ^6^ Department of Pathology Vienna General Hospital, Medical University of Vienna Vienna Austria; ^7^ Department of Biomedical Imaging and Image‐Guided Therapy, Division of Nuclear Medicine Christian Doppler Laboratory for Applied Metabolomics Vienna Austria; ^8^ Unit of Laboratory Animal Pathology University of Veterinary Medicine Vienna Austria; ^9^ Department of Molecular Biology Umeå University Umeå Sweden; ^10^ Center for Biomarker Research in Medicine (CBmed) Graz Austria

**Keywords:** advanced disease, head and neck cancer, immune‐checkpoint therapy, sinonasal squamous cell carcinoma

## Abstract

**Introduction:**

Sinonasal squamous cell carcinoma (SNSCC) is a rare malignancy with limited data on effective treatment modalities in the recurrent and/or metastatic (r/m) setting. While immune checkpoint inhibitors (ICIs) have shown promise in treating head and neck cancers, in general, their effects in SNSCC remain poorly understood. Furthermore, SNSCC patients are frequently excluded from clinical trials, limiting the evidence base for ICI efficacy in this specific subgroup. Therefore, our study evaluated the efficacy and safety of single‐agent ICI therapy in r/m SNSCC.

**Methods:**

We conducted a retrospective multicenter analysis of all r/m SNSCC patients treated with single‐agent ICIs from July 2018 to December 2023 at two tertiary reference centers.

**Results:**

A total of 18 patients received either Pembrolizumab (*n* = 8) or Nivolumab (*n* = 10) for r/m SNSCC. The overall response rate (ORR) to immunotherapy was 11.1% (2/18), with a disease control rate (DCR) of 27.8% (5/18) and a mean PFS and OS of 11.7 (95% CI: 2.3–21.0) months and 18.9 (95% CI: 8.3–29.5) months respectively. Two (11.1%) immune‐related adverse events led to treatment discontinuation. Univariable analysis revealed high pathological grading (*p* = 0.049) as a negative prognostic factor for PFS. In an exploratory comparison with a larger cohort of 121 patients with r/m SCC of the larynx, oropharynx, hypopharynx, or oral cavity receiving ICI therapy, outcomes in SNSCC appeared broadly similar, with no statistically significant differences in PFS (*p* = 0.153), OS (*p* = 0.152), ORR (*p* = 0.401), or DCR (*p* = 0.359).

**Conclusion:**

Immunotherapy may represent a treatment option for patients with SNSCC. Given the limited sample size, these results should be interpreted with caution. Our findings highlight the urgent need to include SNSCC patients in future prospective trials to clarify the role of immunotherapy in this underrepresented population.

## 1 Introduction

Malignancies of the paranasal sinuses are rare comprising 3%–5% of malignancies in the head and neck region. They are characterized by heterogeneous biological and histological features [1, 2]. Currently, over 18 different histological subtypes are classified in the 5th edition of the WHO classification of head and neck tumors [2]. Among those, the squamous cell carcinoma of the paranasal sinuses (SNSCC) is the most common cancer comprising 42%–52% of all reported sinonasal cancer types [2]. While studies report an average annual decline in incidence of approximately 2% over the last 30 years [1], both the 5‐year overall survival and recurrence rate of 50% have remained unchanged [3].

Sinonasal cancers are often diagnosed in advanced stage due to their unspecific primary symptoms [4]. This necessitates advanced‐stage treatment strategies primarily involving surgery and/or radiotherapy [3, 5]. Notably, current treatment modalities in the recurrence and/or metastatic (r/m) setting are limited and are targeted in a multimodal approach with no widely used prognostic markers [6]. While the survival of patients with r/m squamous cell carcinoma (SCC) of the oral, oropharyngeal, hypopharyngeal, and laryngeal region underwent a paradigm shift in recent years with the emergence of new treatment modalities [7, 8], there is still a paucity of data regarding treatment options for r/m SNSCC [7, 8].

Immunotherapy is the current standard of care for patients with r/m head and neck squamous cell carcinomas (HNSCC). In 2016 and 2019, nivolumab and pembrolizumab, two antibodies targeting the PD‐1/PD‐L1 immune checkpoint axis, received FDA approval for first‐line single‐agent treatment for platinum‐refractory disease or r/m HNSCC, respectively [7, 8]. Current studies report that an estimated 13% to 19% of patients with PD‐L1‐expressing tumors respond to immunotherapy [9]. However, SNSCC are usually excluded from major clinical trials regarding ICI treatment, due to their rarity and molecular heterogeneity [7, 8]. Consequently, evidence on ICI efficacy in SNSCC is limited to case reports and small case series [10, 11], resulting in a paucity of data on response rates and adverse events, as underscored by a recent consensus statement [1]. This lack of data is further exacerbated by the emerging subtyping and variable prognoses within SNSCC, emphasizing an urgent need to improve r/m SNSCC management.

Therefore, our study aimed to evaluate treatment response in patients with r/m SNSCC receiving monotherapy with ICI therapy at two tertiary referral centers. A comparative analysis with patients receiving ICI for HNSCC was conducted, assessing differences in response rates, adverse events, and survival outcomes.

## 2 Methods

### 2.1 Study Population

We included all patients between July 2018 and December 2023 who received single‐agent ICI therapy for r/m SNSCC at the Vienna General Hospital (Medical University of Vienna) and the University Hospital Würzburg. For comparative analysis we included consecutive patients treated with pembrolizumab or nivolumab as first or any line for the oral cavity, larynx, oropharynx, and hypopharynx at the Vienna General Hospital. Exclusion criteria were the unavailability of all patient characteristics and demographics prior to treatment initiation. Patients with newly diagnosed, untreated SNSCC were excluded, as these cases are typically managed with curative‐intent multimodal therapy.

### 2.2 Study Design

Baseline characteristics and patient demographics were assessed and retrospectively obtained from electronic medical records. These included age, gender, primary tumor site, HPV status, grading, staging, extent of disease, anti‐PD‐1 inhibitor regimen, line of anti‐PD‐1 inhibitor, combined positive score (CPS), Eastern Cooperative Oncology Group Performance Status (ECOG‐PS), and prior and subsequent palliative systemic therapy. Staging was performed according to the UICC 7th edition [12]. Oligometastatic disease was defined according to the ESTRO–ASTRO consensus as a state of limited metastatic spread with 1–5 metastatic lesions. Primary tumor control was considered optional, consistent with clinical practice in r/m HNSCC [13, 14]. If there was a history of tobacco use or an active smoking habit, patients were classified as smokers.

The primary outcome of the study was progression‐free survival (PFS), with overall survival (OS), objective response rate (ORR) and disease control rate (DCR) as secondary outcomes. PFS was defined as the time from immunotherapy initiation to disease progression or death, while OS was defined as the time from the start of immunotherapy to death. Patients were censored if lost to follow‐up. The best overall response (bOR) was evaluated using CT and/or MRI studies according to RECIST version 1.1 [15] and defined as complete response (CR), partial response (PR), stable disease (SD), or progressive disease (PD). ORR was defined as CR or PR. The DCR was defined as the combined number of patients achieving CR, PR, or SD. Immune‐related adverse events (irAE) were graded according to current guidelines on the management of irAE from Grade 1 to 4 with increasing severity [16]. A Grade 3 toxicity warrants suspension of ICI therapy and initiation of systemic corticosteroids [16]. The study was approved by the Ethics Committee of the Medical University of Vienna (EK 1539/2025) and the Ethical Committee of the University Hospital of Würzburg (EK 20240805‐01).

### 2.3 Statistical Analysis

All graphical and statistical analyses were performed using STATA (version 18.5, available at https://www.stata.com/). Categorical and continuous variables are presented as the number of patients (*n*) and percentages (%) or as minimum/maximum values and standard deviation, respectively. The Kaplan–Meier estimator was used to generate survival curves. To examine the relationship between patient characteristics and PFS/OS, we utilized the univariate Cox proportional hazards model. Due to the limited sample size, multivariable analysis was not performed. Differences in categorical patient data were analyzed using Pearson's chi‐squared test. Fisher's exact test was employed for groups with one or more cell counts below five. A *p*‐value of ≤ 0.05 was considered statistically significant.

## 3 Results

### 3.1 Patient Characteristics

All patient characteristics are shown in Table 1. A total of 18 patients with SNSCC were included in the analysis. The median age was 65 years (range 43–85 years), with 15 (83.3%) males and 3 (16.7%) females. Tumor localization was predominantly in the nasal cavity (50%) and maxillary sinus (38.9%), while laterality was mainly unilateral (83.3%) with two patients presenting with bilateral tumor (16.7%). All patients presented with advanced‐stage disease (Stage IV); 38.9% were stage IVC, 33.3% were stage IVB, and 22.2% were stage IVA, while in one patient staging was not reported. Disease status at treatment initiation was primarily locoregional (55.6%), followed by locoregional and metastatic disease (38.9%), with only 5.6% having purely metastatic disease. However, all patients did not meet criteria for oligometastatic disease [13, 14]. Among those with metastatic spread, all had ≥ 5 metastatic lesions, predominantly in the lung (87%). One (13%) patient had ≥ 5 bone and hepatic metastatic lesions. HPV status was unknown in 38.9% of patients, negative in 44.4%, and positive in 16.7%. PD‐L1 status (CPS ≥ 1) was positive in 27.8%, negative in 22.2%, and unknown in 50% of patients. Tumor grade was most commonly grade 2 (55.6%). ECOG‐PS was equally distributed between 0 and 1 (44.4% each). Treatment with anti‐PD‐1 inhibitors consisted primarily of nivolumab (55.6%), with pembrolizumab used in 44.4% of cases. In total 8 (44.4%) and 4 (22.2%) patients received therapy due to platinum‐refractory disease and cetuximab refractory disease respectively. Prior systemic palliative chemotherapy was administered to 33.3% of patients, and subsequent chemotherapy regimens were administered to 44.4% of patients.

**TABLE 1:** Patient and treatment characteristics of SNSCC patients treated with ICI therapy.

	Sinonasal
	*n* (%)
Total	18 (100.0%)
Age	
< 65 years	11 (61.1%)
≥ 65 years	7 (38.9%)
Gender	
Male	15 (83.3%)
Female	3 (16.7%)
Smoking	
Current or former	11 (61.1%)
Never	3 (16.7%)
Not reported	4 (22.2%)
Localization	
Maxillary Sinus	7 (38.9%)
Nasal Cavity	9 (50.0%)
Other	2 (11.1%)
Laterality	
Left	8 (44.4%)
Right	7 (38.9%)
Bilateral	3 (16.7%)
Stage	
Not reported	1 (5.6%)
IVA	4 (22.2%)
IVB	6 (33.3%)
IVC	7 (38.9%)
Disease status	
Locoregional	10 (55.6%)
Metastatic	1 (5.6%)
Locoregional and metastatic	7 (38.9%)
HPV status	
Negative	8 (44.4%)
Positive	3 (16.7%)
Unknown	7 (38.9%)
PD‐L1 status (CPS ≥ 1)	
Negative	4 (22.2%)
Positive	5 (27.8%)
Unknown	9 (50.0%)
Grade	
1	1 (5.6%)
2	10 (55.6%)
3	3 (16.7%)
Unknown	4 (22.2%)
ECOG‐PS	
0	8 (44.4%)
1	8 (44.4%)
≥ 2	2 (11.1%)
Anti‐PD‐1 inhibitor	
Nivolumab	10 (55.6%)
Pembrolizumab	8 (44.4%)
Platinum sensitivity	
Sensitive	10 (55.6%)
Refractory	8 (44.4%)
Cetuximab sensitivity	
Sensitive	14 (77.80%)
Refractory	4 (22.2%)
Prior systemic palliative CHT	
0	12 (66.7%)
≥ 1	6 (33.3%)
Subsequent CHT regiments	
0	10 (55.6%)
≥ 1	8 (44.4%)

*Note:* § = frontal sinus (*n* = 1) and sphenoidal sinus (*n* = 1).

Abbreviations: CHT, chemotherapy; CPS, combined positive score; ECOG‐PS, Eastern Cooperative Oncology Group Performance Status; HPV, human papilloma virus.

### 3.2 Treatment Response, Adverse Events and Survival

**FIGURE 1:**
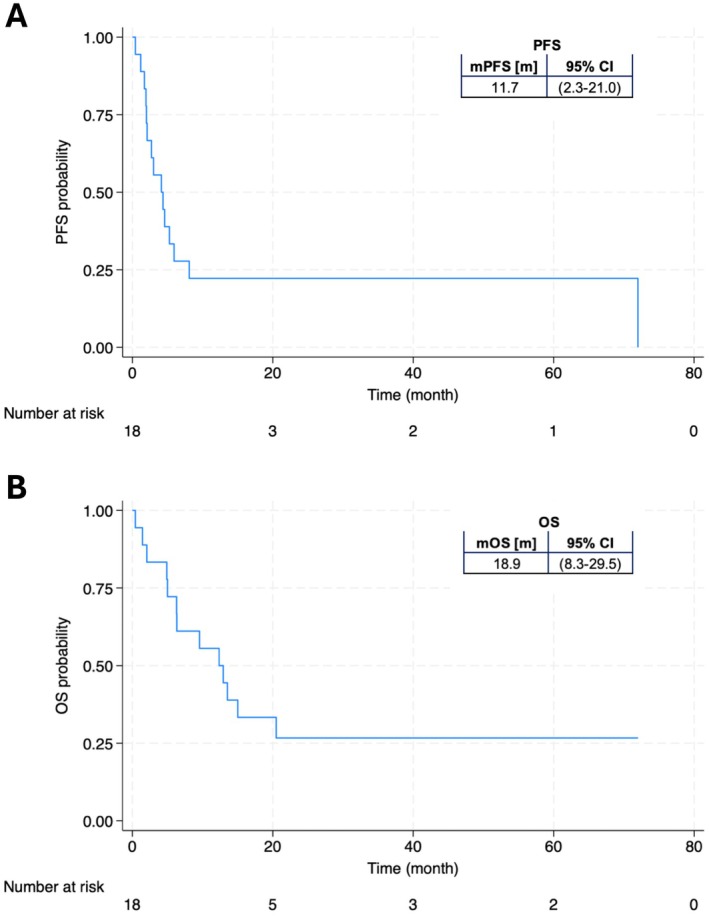
Kaplan Meier estimator of (A) PFS and (B) OS in SNSCC patients treated with ICI therapy. CI, confidence interval; m, months; OS, overall survival; PFS, progression‐free survival.

Treatment response and adverse events are shown in Table 2. Analysis of bOR revealed that two patients (11.1%) achieved a CR. No PRs were observed. SD was noted in three patients (16.7%), while PD occurred in 13 patients (72.2%). Regarding irAEs, 16 patients (88.9%) experienced none or any grade < 3, and only two patients (11.1%) experienced a Grade 3 irAE. The ORR and DCR were 11.1% and 27.8%, respectively. There was no significant difference in bOR (*p* = 0.294) or the incidence of irAE (*p* = 0.477) between patients treated with Nivolumab and those treated with Pembrolizumab. Figure 1 shows Kaplan–Meier survival estimator of PFS and OS of the SNSCC patient cohort. The observed mPFS and mOS were 11.7 months (95% CI: 2.3–21.0; Figure 1A) and 18.9 months (95% CI: 8.3–29.5; Figure 1B), respectively.

**TABLE 2:** bOR and irAE Grade stratified by regiment.

	Total	Pembrolizumab	Nivolumab	*p*
	*n* (%)	*n* (%)	*n* (%)	
bOR				
CR	2 (11.1%)	2 (25.0%)	0 (0.0%)	
PR	0 (0.0%)	0 (0.0%)	0 (0.0%)	
SD	3 (16.7%)	0 (0.0%)	3 (30.0%)	
PD	13 (72.2%)	6 (75.0%)	7 (70.0%)	0.294
irAE Grade				
< 3	16 (88.9%)	8 (64.5%)	8 (57.1%)	
≥ 3	2 (11.1%)	0 (0.0%)	2 (14.3%)	0.477
ORR	2 (11.1%)	2 (25.0%)	0 (0.0%)	0.183
DCR	5 (27.8%)	2 (25.0%)	3 (30.0%)	0.618

*Note:* Distribution of both groups was analyzed using Chi‐squared or Fisher's exact test.

Abbreviations: bOR, best overall response; CR, complete response; DCR, disease control rate; irAE, immune‐related adverse events; ORR, objective response rate; PD, progressive disease; PR, partial response; SD, stable disease.

### 3.3 Univariable Analysis

Results of the univariable analysis for PFS and OS are shown in Table 3. Female patients were associated with a higher risk of worse OS (HR = 9.3, 95% CI: 1.0–57.5, *p* = 0.017). This was also observed for tumors located in “Other” sites compared to maxillary sinus tumors (HR = 10.1, 95% CI: 1.2–83.9, *p* = 0.033). Additionally, Grade 3 tumors were linked to a significantly higher risk of disease progression (HR = 4.3, 95% CI: 1.0–18.2, *p* = 0.049).

**TABLE 3:** Univariable analysis of PFS and OS for the SNSCC cohort. Statistically significant results are shown in bold print.

	PFS			OS		
	HR	95% CI	p	HR	95% CI	p
Age						
< 65 years	Ref			Ref		
≥ 65 years	0.4	(0.1–1.4)	0.154	0.4	(0.1–1.5)	0.184
Gender						
Male	Ref			Ref		
Female	3.4	(0.8–14.3)	0.098	9.3	(1.5–57.5)	**0.017**
Localization						
Maxillary Sinus	Ref			Ref		
Nasal Cavity	1.3	(0.4–4.1)	0.657	1.3	(0.4–4.2)	0.699
Other	4.6	(0.8–27.8)	0.096	10.1	(1.2–83.9)	**0.033**
Stage (*n* = 17)						
IVA	Ref			Ref		
IVB	0.3	(0.1–1.2)	0.09	0.6	(0.1–2.6)	0.484
IVC	0.3	(0.1–1.1)	0.072	0.5	(0.1–2.2)	0.368
Disease status						
Recurrent	Ref			Ref		
Metastatic	2.3	(0.3–20.7)	0.454	0.9	(0.1–7.1)	0.886
Recurrent and metastatic	0.6	(0.2–1.9)	0.389	0.8	(0.2–2.5)	0.681
HPV status (*n* = 11)						
Negative	Ref					
Positive	1.4	(0.3–6.8)	0.708	1.2	(0.2–6.2)	0.837
PD‐L1 status (CPS ≥ 1) (*n* = 9)						
Negative	Ref					
Positive	0.8	(0.2–3.9)	0.772	0.5	(0.1–2.6)	0.403
Grade (*n* = 14)						
< 3	Ref			Ref		
3	4.3	(1.0–18.2)	**0.049**	1.4	(0.3–6.6)	0.676
ECOG‐PS						
< 2	Ref			Ref		
≥ 2	0.9	(0.5–1.6)	0.636	1.1	(0.6–2.1)	0.72
Anti‐PD‐1 inhibitor						
Pembrolizumab	Ref			Ref		
Nivolumab	2	(0.7–5.9)	0.233	1.5	(0.5–4.5)	0.516
Platinum sensitivity						
Sensitive	Ref			Ref		
Refractory	1	(0.4–3.0)	0.929	0.7	(0.2–2.1)	0.525
Cetuximab sensitivity						
Sensitive	Ref					
Refractory	1.9	(0.57–6.1)	0.305	2.2	(0.7–7.1)	0.196
Prior systemic palliative CHT						
0	Ref			Ref		
≥ 1	1.7	(0.6–4.9)	0.327	2.3	(0.9–7.0)	0.13
Subsequent CHT regiments						
0	Ref			Ref		
≥ 1	1.4	(0.5–4.1)	0.537	0.8	(0.3–2.3)	0.633

*Note:* § = frontal sinus (*n* = 1) and sphenoidal sinus (*n* = 1).

Abbreviations: CHT, chemotherapy; CI, confidence interval; CPS, combined positive score; ECOG‐PS, Eastern Cooperative Oncology Group Performance Status; HPV, human papilloma virus; HR, hazard ratio; m, months; OS, overall survival; PFS, progression‐free survival.

### 3.4 Comparison With Consecutive Patient Cohort

A cohort of 121 consecutive patients with oral, oropharyngeal, hypopharyngeal or laryngeal cancer who received ICI therapy at the Vienna General Hospital (Medical University of Vienna) between July 2018 and December 2023 was analyzed. All *p*‐values reported below represent comparisons to the sinonasal cohort. Patient characteristics are detailed in Table 4. The cohort included 58 (47.9%) patients under 65 years and 63 (52.1%) aged 65 years or older (*p* = 0.324). Male patients comprised 82 (67.8%) of the cohort (*p* = 0.271). Smoking history revealed 89 (73.6%) current or former smokers and 16 (13.2%) never‐smokers (*p* = 0.696). ECOG‐PS showed no significant difference between groups (*p* = 0.595). HPV status (*p* = 1.0) and PD‐L1 positivity (*p* = 0.568) also showed no significant differences. Disease extent at the initiation of ICI therapy showed 52 (43.0%) patients with locoregional, 16 (13.2%) with metastatic disease, and 53 (43.8%) with both locoregional and metastatic disease (*p* = 0.599). Nivolumab was administered to 77 (63.6%) patients, while pembrolizumab was used in the remainder (*p* = 0.129). ICI therapy served as first‐line palliative treatment for 86 (71.1%) patients (*p* = 0.783); 35 (28.9%) received prior systemic chemotherapy, and 47 (38.8%) received subsequent chemotherapy (*p* = 0.783 and *p* = 0.425, respectively). There was no difference between both cohorts regarding platinum and cetuximab sensitivity between both groups (*p* = 0.432 and *p* = 0.423).

**TABLE 4:** Patient demographics and characteristics dichotomized in sinonasal and oral–pharyngeal–laryngeal subsite.

	Sinonasal	Oral–Pharyngeal–Laryngeal	*p*
	*n* (%)	*n* (%)	
Age			
< 65 years	11 (61.1%)	58 (47.9%)	
≥ 65 years	7 (38.9%)	63 (52.1%)	0.324
Gender			
Male	15 (83.3%)	82 (67.8%)	
Female	3 (16.7%)	39 (32.2%)	0.271
Smoking			
Current or former	11 (61.1%)	89 (73.6%)	
Never	3 (16.7%)	16 (13.2%)	0.696
Not reported	4 (22.2%)	16 (13.2%)	
Disease status			
Locoregional	10 (55.6%)	52 (43.0%)	
Metastatic	1 (5.6%)	16 (13.2%)	
Locoregional and metastatic	7 (38.9%)	53 (43.8%)	0.599
HPV status			
Positive	8 (44.4%)	69 (57.0%)	
Negative	3 (16.7%)	24 (19.8%)	1
Unknown	7 (38.9%)	28 (23.1%)	
PD‐L1 status (CPS ≥ 1)			
Positive	4 (22.2%)	68 (56.2%)	
Negative	5 (27.8%)	2 (1.7%)	0.568
Unknown	9 (50.0%)	51 (42.1%)	
Grade			
1	1 (5.6%)	2 (1.7%)	
2	10 (55.6%)	89 (73.6%)	
3	3 (16.7%)	22 (18.2%)	0.333
Unknown	4 (22.2%)	8 (6.6%)	
ECOG‐PS			
0	8 (44.4%)	47 (38.8%)	
1	8 (44.4%)	41 (33.9%)	
≥ 2	2 (11.1%)	33 (27.3%)	0.595
Anti‐PD‐1 inhibitor			
Nivolumab	10 (55.6%)	77 (63.6%)	
Pembrolizumab	8 (44.4%)	44 (36.4%)	0.129
Platinum sensitivity			
Sensitive	10 (55.6%)	80 (66.1%)	
Refractory	8 (44.4%)	41 (33.9%)	0.432
Cetuximab sensitivity			
Sensitive	14 (77.8%)	80 (66.1%)	
Refractory	4 (22.2%)	41 (33.9%)	0.423
Prior systemic palliative CHT			
0	12 (66.7%)	86 (71.1%)	
≥ 1	6 (33.3%)	35 (28.9%)	0.783
Subsequent CHT regiments			
0	10 (55.6%)	74 (61.2%)	
≥ 1	8 (44.4%)	47 (38.8%)	0.425

*Note:* Distribution of both groups was analyzed using Chi‐squared or Fisher's exact test.

Abbreviations: CHT, chemotherapy; CPS, combined positive score; ECOG‐PS, Eastern Cooperative Oncology Group Performance Status; HPV, human papilloma virus.

### 3.5 Comparison of Survival and Treatment Response

**FIGURE 2:**
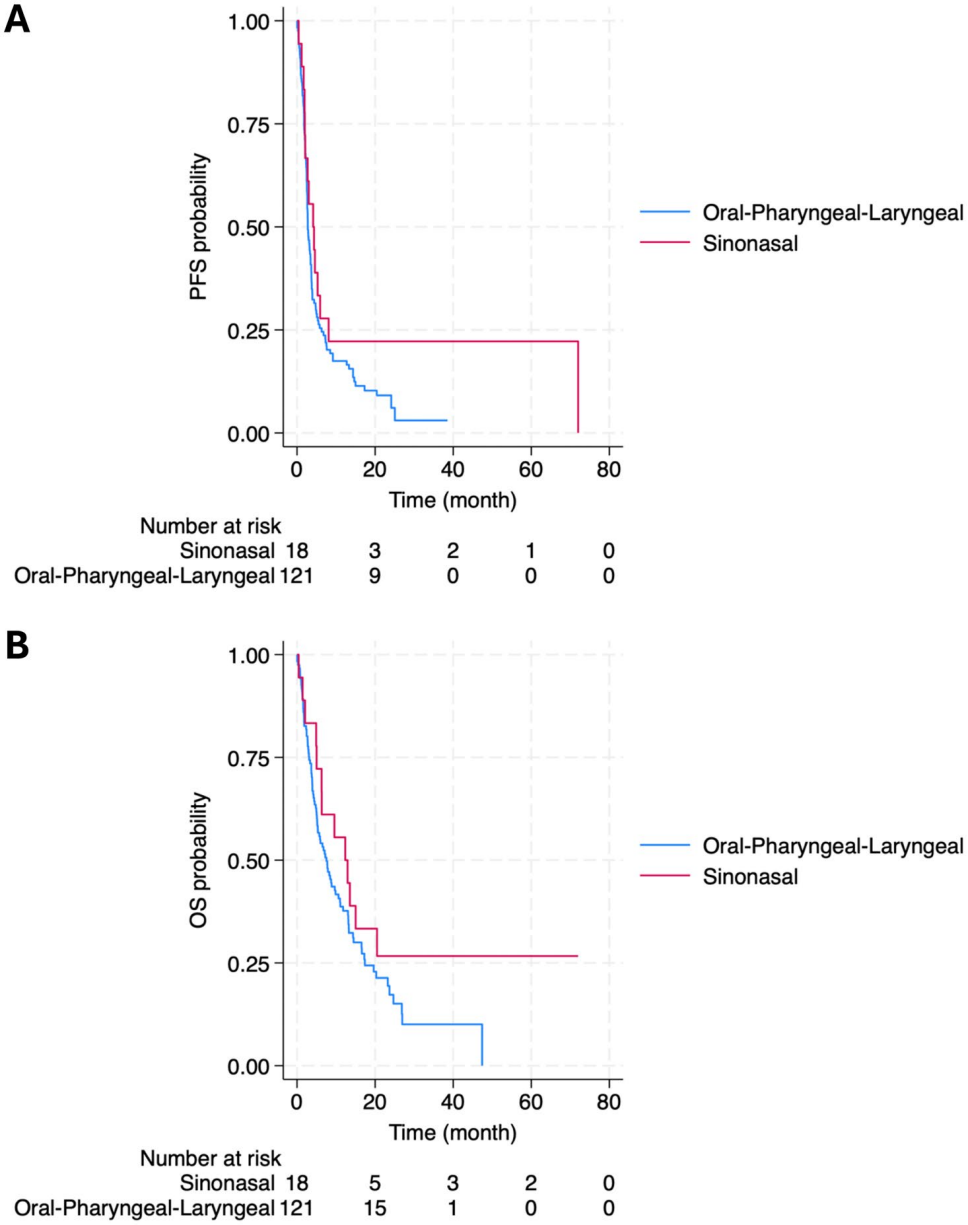
Kaplan Meier estimator of (A) PFS and (B) OS stratified by primary site. m, months; OS, overall survival; PFS, progression‐free survival.

Treatment response data are shown in Table 5. The bOR was similar between groups (*p* = 0.177). In the oral–pharyngeal–laryngeal group, 7 (5.8%) achieved CR, 20 (16.5%) achieved PR, 16 (13.2%) had SD, and 78 (64.5%) showed PD. In the sinonasal group, 2 (11.1%) achieved CR, none had PR, 3 (16.7%) had SD, and 13 (72.2%) experienced PD. The ORR was 22.3% in the oral–pharyngeal–laryngeal group and 11.1% in the sinonasal group (*p* = 0.401). The DCR was 35.5% in the oral–pharyngeal–laryngeal group and 27.8% in the sinonasal group (*p* = 0.359). Moreover, there was no difference in irAE between both groups which led to treatment discontinuation (*p* = 1.000). Kaplan Meier estimators of oral–pharyngeal–laryngeal and sinonasal subsites are shown in Figure 2. There was no significant difference in mPFS between both groups (5.5 months, 95% CI: 4.3–6.7 vs. 11.7 months, 95% CI: 2.3–21.0) (HR = 0.66, *p* = 0.153) (Table 6). This was also observed for OS, with a mOS of 9.5 months (95% CI: 8.0–11.1) and 18.9 months (95% CI: 8.3–29.5) for both groups respectively (HR = 0.65, *p* = 0.153) (Table 6).

**TABLE 5:** bOR and irAE rate of sinonasal compared to oral–pharyngeal–laryngeal subsite. Difference between bOR was compared using Fisher's exact test.

	Sinonasal	Oral–Pharyngeal–Laryngeal	*p*
	*n* (%)	*n* (%)	
bOR			
CR	2 (11.1%)	7 (5.8%)	
PR	0 (0.0%)	20 (16.5%)	
SD	3 (16.7%)	16 (13.2%)	
PD	13 (72.2%)	78 (64.5%)	0.177
irAE Grade			
< 3	16 (88.9%)	105 (86.8%)	
≥ 3	2 (11.1%)	16 (13.2%)	1
ORR	2 (11.1%)	27 (22.3%)	0.401
DCR	5 (27.8%)	43 (35.5%)	0.359

Abbreviations: BOR, best overall response; CR, complete response; DCR, disease control rate; irAE, immune‐related adverse events; ORR, objective response rate; PD, progressive disease; PR, partial response; SD, stable disease.

**TABLE 6:** Survival and univariable Cox regression stratified by primary site.

	PFS				OS			
	mPFS (m)	95% CI	HR	*p*	mOS (m)	95% CI	HR	*p*
Total	6.3	(4.8–7.9)			10.8	(8.8–12.7)		
Oral–Pharyngeal–Laryngeal	5.5	(4.3–6.7)	Ref	Ref	9.5	(8.0–11.1)	Ref	Ref
Sinonasal	11.7	(2.3–21.0)	0.66	0.153	18.9	(8.3–29.5)	0.65	0.152

Abbreviations: m, months; OS, overall survival; PFS, progression‐free survival; ref., reference.

## 4 Discussion

While immunotherapy has become the standard of care for r/m HNSCC, SNSCC patients have often been excluded from major clinical trials due to the rarity and heterogeneity of this malignancy. Our findings show that ICI monotherapy is a viable treatment option for SNSCC, demonstrating moderate efficacy with outcomes comparable to those observed in oral, pharyngeal, and laryngeal SCC.

To the best of our knowledge, we report on the largest cohort investigating ICI therapy in SNSCC. The observed ORR of 11.1% and DCR of 27.8% are consistent with the limited data available on ICI therapy in SNSCC. Park et al. reported on 11 SNSCC patients receiving ICI therapy, observing a mPFS of 4.2 months and ORR of 27.2% [11]. While their ORR is comparable to our cohort, their PFS is significantly lower than the observed 11.7 months in the present cohort [11]. Kim et al. studied 11 patients with nasal cavity and paranasal sinus cancer treated with ICIs, reporting an ORR of 18.2% and a median PFS of 3 months [17]. Interestingly, despite 5 of 11 patients achieving SD, their short PFS raises questions about the robustness of these SD definitions [17]. A recent study by Ueda et al. investigated every excluded patient of the Checkmate 141 trial. The authors included 18 patients with malignancies of the sinonasal cavity; however, with multiple histological entities including 11 SCC [18]. Overall, they observed a 6.7% ORR, a 46.7% DCR, a mPFS of 2.5 months and a mOS of 27.7 months. However, due to the inhomogeneous cohort, treatment outcomes could not be extracted for SCC patients. Interestingly, our study revealed a nearly doubled PFS and OS of the sinonasal compared to the oral–pharyngeal–laryngeal patients and data from large clinical trials. These unexpectedly long median PFS and OS may largely reflect patient selection. First, none of the patients met oligometastatic criteria. However, most patients had metastases confined to a single organ, predominantly the lung, a pattern reported to be associated with better outcome [13, 14, 19]. Most importantly, two patients in our cohort achieved exceptionally long survival exceeding 2000 days. Such outcomes, while rare, are consistent with the well‐described ‘tail of the curve’ phenomenon observed with ICI therapy, where a subset of patients derive sustained benefit and survival curves reach a plateau [20, 21]. In conclusion, the presence of exceptional responders and the small cohort size likely contributed to the longer survival estimates compared to historical r/m HNSCC cohorts. These findings emphasize the exploratory nature of our results and the need for cautious interpretation.

A significant challenge with available studies on ICI treatment in SNSCC is limited comparison by variations in study designs and patient characteristics. Notably, the Checkmate 141 trial focused on therapy response in a platinum‐resistant setting, whereas our study included a mixed cohort of both platinum‐resistant and platinum‐sensitive patients, which may explain the differing results. However, the difference was not associated with worse OS and PFS in our cohort. Nevertheless, our study contributes to the growing body of literature demonstrating comparable response rates and survival in r/m SNSCC.

In the r/m HNSCC, established prognostic factors include the ECOG‐PS and the CPS ≥ 1for PD‐L1 expression [7, 8]. However, in the present study, only female sex and tumor grading demonstrated significant prognostic value. Notably, poorly differentiated tumors are typically associated with worse survival outcomes in HNSCC [22]. These findings should be interpreted with caution due to the limited sample size of the present study.

In this study, two patients (11.1%) experienced a Grade 3 irAE, both presenting with an exanthema requiring intravenous corticosteroids. In comparison, there was no significant difference in the irAE rate in the oral, pharyngeal, laryngeal cohort. Landmark trials of monotherapy ICIs reported irAE rates of 13.1% for nivolumab and 55% for pembrolizumab, with 8% irAEs resulting in fatal outcomes in the Keynote‐048 trial. The findings of the present study suggest that ICI monotherapy can be safely applied in patients with SNSCC, with a manageable irAE profile.

A limitation of our study is its limited scope, failing to encompass the significant molecular diversity within SCC of paranasal sinuses [2]. This heterogeneity includes various subtypes like SWI/SNF complex‐deficient (characterized by aggressive growth) and HPV‐associated (despite aggressive histology, often showing a favorable prognosis) tumors among other [2]. Specifically, the DEK::AFF2 fusion SCC, a subtype of non‐keratinizing sinonasal SCC, which, despite limited data, including a case report of exceptional response to pembrolizumab, requires further investigation [23, 24, 25]. Moreover, while the impact of these subtypes on immunotherapy outcomes remains largely unknown, other sinonasal cancer entities, such as SNUC, have shown favorable immune profiles in transcriptomic analyses, potentially justifying immunotherapy use [26]. This suggests that despite the complexity, there is potential for identifying favorable subtypes, for targeted immunotherapy approaches.

In the present study we observed two patients with CR. The first patient, a 77‐year‐old with T3N0M0 HNSCC underwent surgery, radiotherapy, and later pembrolizumab for recurrence with metastases. By 28 cycles, CR was achieved, pembrolizumab was stopped and remission remained until non‐disease‐related death at 68 months post ICI start. The second patient, an 80‐year‐old with locoregionally advanced T4bN2bM0 SNSCC, who declined primary chemoradiotherapy, started primary pembrolizumab, achieved CR, and remains alive with ongoing treatment after 15 months. The increasing number of case reports detailing CRs to ICIs in sinonasal cancers is a key consideration in determining their standard‐of‐care role. These reports demonstrate potential efficacy beyond SNSCC, encompassing cases responding after fulminant irAEs [10], SMARCB1‐deficient cancer achieving CRs [27], and even successful CRs with neoadjuvant tislelizumab [28]. Moreover, CRs have also been observed in similar malignancies like SNUC [29] treated with various combinations of ICIs, including ipilimumab and COX2 inhibitors [30], and in NUT midline carcinoma treated with nivolumab and BET inhibitors [31]. While this promising data suggests a broader therapeutic potential, caution is warranted by the documented case of hyperprogression, highlighting the need for rigorous clinical trials and careful patient selection [32].

### 4.1 Limitations

Our study comes with inherent limitations. First, the retrospective nature of this study and the small sample size limit the generalizability of our findings. Most importantly, the small sample size leads to restricted statistical power and results in wide confidence intervals, particularly for survival analyses. Consequently, comparisons with larger HNSCC cohorts lack robustness, and the results should be regarded as exploratory rather than definitive. Moreover, the small sample size precluded multivariate analysis. As a result, we cannot exclude potential confounding effects of prior therapies, PD‐L1 status, and other clinical factors on treatment response, which further limits the strength of our conclusions. Additionally, the inclusion of only two tertiary centers may not fully capture the diversity of SNSCC cases seen in clinical practice. Finally, the lack of comprehensive molecular profiling in our dataset prevents us from identifying potential molecular biomarkers that could predict response to immunotherapy.

Despite these limitations, our study underscores the importance of including SNSCC patients in future clinical trials of ICI therapy to address the current evidence gap. Ongoing trials like I‐NAPA (NCT05027633), which investigates immunotherapy with chemotherapy and chemoradiation for advanced SCC of the nasal cavity and paranasal sinuses, will provide valuable insights that may translate to improved SNSCC management [33].

### 4.2 Conclusion

In conclusion, this study provides preliminary evidence that monotherapy with ICIs may offer clinical benefit in selected patients with r/m SNSCC. However, the small sample size, heterogeneity, and wide confidence intervals necessitate cautious interpretation, and the findings should be regarded as exploratory. Prospective trials and molecularly informed studies are essential to determine the true efficacy of ICI therapy in this rare and underrepresented population.

## Author Contributions

**Alexander Lein:** conceptualization (equal), data curation (equal), formal analysis (equal), investigation (equal), visualization (equal), writing – original draft (equal). **Thorsten Fuereder:** methodology (equal), validation (equal), writing – review and editing (equal). **Manuel Stoeth:** conceptualization (equal), formal analysis (equal), methodology (equal), validation (equal), writing – original draft (equal), writing – review and editing (equal). **Agmal Scherzad:** investigation (equal), methodology (equal), validation (equal), writing – review and editing (equal). **Stephan Hackenberg:** formal analysis (equal), supervision (equal), writing – review and editing (equal). **Julia Schnöll:** writing – original draft (equal), writing – review and editing (equal). **Lorenz Kadletz‐Wanke:** writing – review and editing (equal). **Gregor Heiduschka:** writing – review and editing (equal). **Archana Jaiswal:** writing – review and editing (equal). **Rajiv Bhalla:** writing – review and editing (equal). **Lukas Kenner:** writing – review and editing (equal). **Faris F. Brkic:** conceptualization (equal), project administration (equal), writing – review and editing (equal).

## Consent

The requirement for written informed consent was waived due to the retrospective study design.

## Conflicts of Interest

Thorsten Fuereder received honoraria from MSD, Merck Darmstadt, Roche, BMS, Accord, Sanofi, Boehringer Ingelheim, Amgen, Pfizer and Takeda, and research grants from Merck and MSD. The other authors declare no conflicts of interest related to the publication of this article.

## Data Availability

The data that support the findings of this study are available on request from the corresponding author. The data are not publicly available due to privacy or ethical restrictions.
